# All-atmospheric fabrication of Ag–Cu core–shell nanowire transparent electrodes with Haacke figure of merit >600 × 10^–3^ Ω^−1^

**DOI:** 10.1038/s41598-022-25080-x

**Published:** 2022-12-05

**Authors:** Steven J. DiGregorio, Collin E. Miller, Kevin J. Prince, Owen J. Hildreth, Lance M. Wheeler

**Affiliations:** 1 National Renewable Energy Laboratory, Golden, CO 80401 USA; 2 Colorado School of Mines, Golden, CO 80401 USA

**Keywords:** Electronic properties and materials, Nanowires, Nanowires, Synthesis and processing

## Abstract

Transparent conducting electrodes (TCEs) are essential components in devices such as touch screens, smart windows, and photovoltaics. Metal nanowire networks are promising next-generation TCEs, but best-performing examples rely on expensive metal catalysts (palladium or platinum), vacuum processing, or transfer processes that cannot be scaled. This work demonstrates a metal nanowire TCE fabrication process that focuses on high performance and simple fabrication. Here we combined direct and plating metallization processes on electrospun nanowires. We first directly metallize silver nanowires using reactive silver ink. The silver catalyzes subsequent copper plating to produce Ag–Cu core–shell nanowires and eliminates nanowire junction resistances. The process allows for tunable transmission and sheet resistance properties by adjusting electrospinning and plating time. We demonstrate state-of-the-art, low-haze TCEs using an all-atmospheric process with sheet resistances of 0.33 Ω sq^−1^ and visible light transmittances of 86% (including the substrate), leading to a Haacke figure of merit of 652 × 10^–3^ Ω^−1^. The core–shell nanowire electrode also demonstrates high chemical and bending durability.

## Introduction

**Figure 1:**
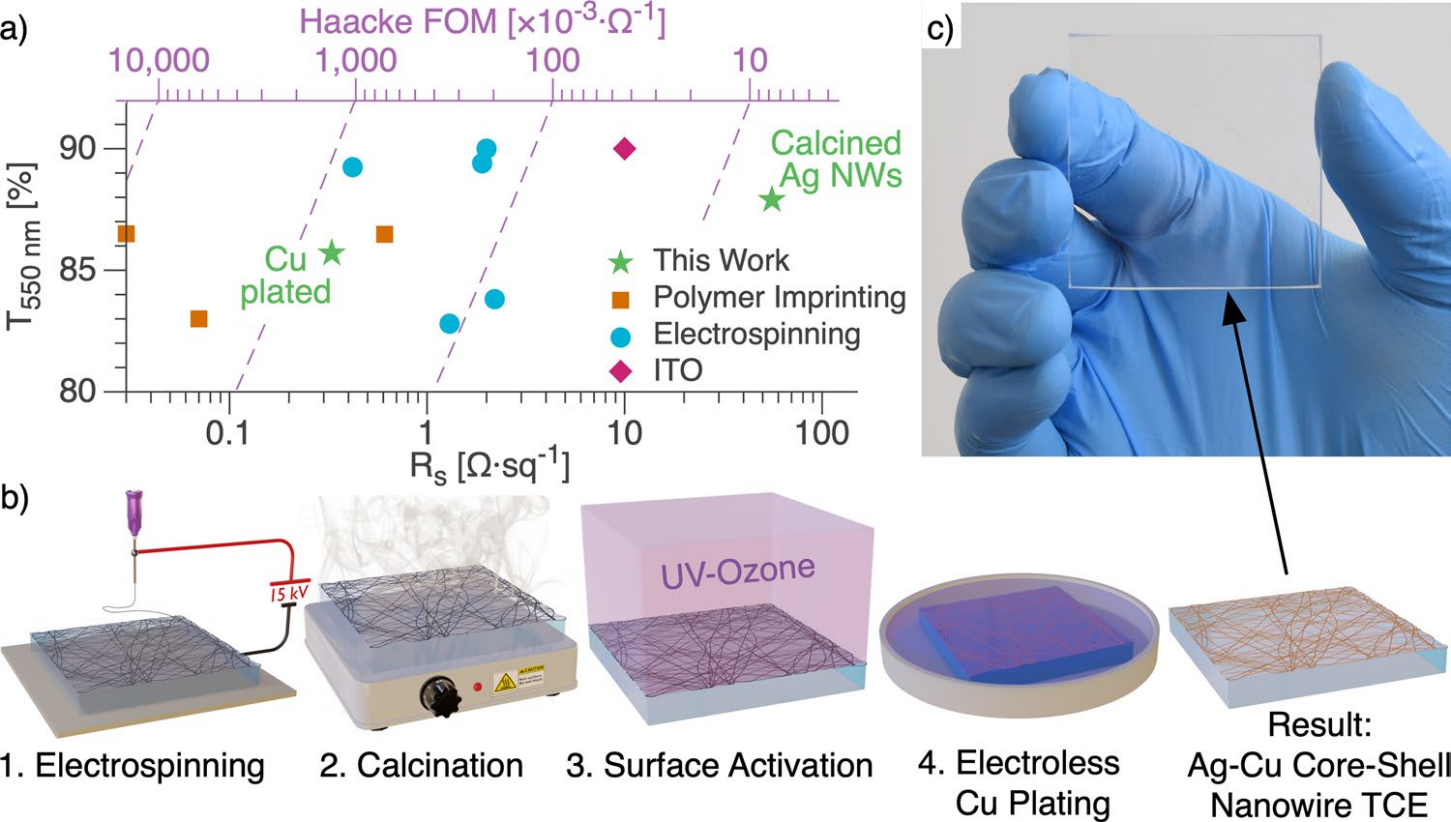
(**a**) Transmission at 550 nm (T_550 nm_) as a function of sheet resistance (R_s_) plot showing the highest performance nanowire TCEs to date. TCEs based on polymer imprinting^22–24^, electrospinning^31–35^, and ITO^2^ are included. The green stars are the highest FOM TCEs from this work for silver nanowires after calcination and silver nanowires with copper plating. The dashed purple lines are Haacke FOM isolines. Some transmittance values were recalculated from their original publications to include comparable substrates (Table S1). (**b**) Schematic of the TCE fabrication process. 1. An electrospinner deposits polymer nanowires that contain reactive silver ink onto a glass substrate. 2. A hotplate at 300 °C calcines the nanowires for 30 s to vaporize the polymer and reduce the silver precursor. 3. A UV-ozone treatment activates the silver nanowire surface for electroless copper deposition. 4. Electroless copper deposition creates a copper shell around the silver nanowires to fuse the junctions. A core–shell silver–copper nanowire network remains on the substrate. (**c**) Photograph showing real-world transparency and color neutrality of nanowire TCE.

Existing and emerging optoelectronic technologies, such as photovoltaics, light-emitting diodes, touch screens, fully transparent displays, and transparent heaters, rely on transparent conducting electrodes (TCEs) to operate. High-performance TCEs optimize two material properties, sheet resistance (R_s_) and light transmittance (T), that are difficult to control independently since both depend on the amount of TCE material; greater amounts of TCE material increase R_s_ and decrease T. The tradeoff between R_s_ and T is a problem for all TCEs. Optimization of this tradeoff is quantified by a figure of merit (FOM) that combines R_s_ and T into a single value. The widely used Haacke FOM is equal to T^10^ R_s_^−1^^1^. Indium Tin Oxide (ITO), the dominant commercial TCE technology, typically achieves R_s_ of ~ 10 Ω sq^−1^, ~ 90% T, and a FOM of around 35 × 10^–3^ Ω^−1^ (Fig. 1a)^2^. In addition to relatively low performance, ITO has several other drawbacks that limit applicability and increases cost^3^: (1) It is brittle in nature, rendering it unsuitable for flexible applications. (2) Indium is increasingly expensive due to its scarcity. (3) ITO deposition usually involves time-consuming and expensive vacuum processes, like magnetron sputtering.

Researchers have developed a number of strategies to displace incumbent ITO, including TCEs based on metal oxides^4,5^, carbon nanotubes^6,7^, graphene^8,9^, conductive polymers^10,11^, oxide/metal/oxide strutures^12–15^, and metal nanowire networks^15–19^. Metal nanowire networks are promising due to their high performance, high scalability, low cost, and flexibility^20,21^. Metals provide unmatched electrical performance, and thin wires are nearly invisible to the human eye at low fill percentages. High-performance metal nanowire TCEs contain low-resistivity wires with uniform distribution and fused junctions to reduce contact resistances. There are various nanowire patterning techniques, including polymer imprinting^22–25^, electrohydrodynamic printing^26,27^, solution processing^28,29^, cracked film lithography^30^, and electrospinning^31–36^. Polymer imprinting and electrospinning are two of the most common patterning methods due to their scalability and low cost. Figure 1a provides a survey of some of the highest performance metal nanowire TCEs based on polymer imprinting and electrospinning.

Chen et al. used polymer imprinting to achieve the highest FOM nanowire TCE to date (~ 7800 × 10^–3^ Ω^−1^)^24^. The process involved imprinting high aspect-ratio micro-grooves into a polymer substrate. A secondary electroplating process metallized the grooves. The TCEs produced exhibit extremely high FOMs but are limited to only polymer substrates due to the imprinting process. Furthermore, these TCEs usually have large wires (~ 5 µm), which leads to haze or visible obstruction and less suitability for aesthetic applications, like windows or displays.

Electrospinning is a simple nanowire fabrication process that does not have the same drawbacks as polymer imprinting while still producing some of the highest performance nanowire TCEs (FOM = 762 × 10^–3^ Ω^−1^)^33^. Electrospinning can deposit nanowires onto many substrate materials and produce fine nanowires (< 200 nm diameter), resulting in low haze. Electrospinning uses strong electric fields to accelerate viscous, polymeric solutions from a metal needle onto a collector plate^37^. The polymer’s viscoelastic properties result in a continuous wire, and the large needle-to-collector distance (~ 10 cm) allows the solvent to evaporate and the wires to draw thin. The result is a randomly distributed network of high-aspect-ratio polymer nanowires.

Two main methods exist for converting electrospun polymer nanowires to conductive metal nanowires: direct metallization^31,32^ and plating^33,38^. Direct metallization involves electrospinning a polymer solution that contains metallic precursors. The resulting nanowires are calcined to vaporize the precursor polymers and sinter metallic particles together. Plating involves electrospinning a polymer solution that contains a catalyst, followed by an electroplating or electroless deposition step to plate metal shells around the polymer nanowires. An et al. utilized plating to fabricate the highest-performing electrospun TCE to date (FOM = 762 × 10^–3^ Ω^−1^)^33^. However, their method is quite involved and includes two electrospinning steps, electrodeposition that relies on a platinum catalyst, and transfer from a non-scalable electrically-conductive frame.

In this work, we take a hybrid direct metallization and plating approach to remove expensive materials and simplify the process to fabricate scalable, state-of-the-art nanowire TCEs. We use a simple 4-step process that consists of electrospinning, calcination, surface activation, and electroless copper deposition (Fig. 1b). First, we electrospin a polymer and reactive silver ink solution onto a clean glass substrate, following the Kiremitler et al. procedure^31^. After electrospinning, a 300 °C hotplate calcines the wires to vaporize the polymers and yield silver nanowires. UV-ozone treatment activates the silver nanowire surface for quicker and more uniform electroless copper deposition. Whereas previous reports use platinum^33^, palladium^38,39^, or ruthenium^40^ catalysts for plating, here, we electrolessly plate copper directly onto the silver nanowires to yield an Ag–Cu core–shell nanowire network. The resultant nanowire network is nearly indistinguishable from bare glass to the naked eye (Fig. 1c). Our highest performing TCEs exhibit a R_s_ = 0.33 Ω sq^−1^ and T of 86% (FOM = 652 × 10^–3^ Ω^−1^). The result is the second-highest FOM electrospun TCE that we are aware of to date (Fig. 1a) and used processes that are more applicable to commercialization. Our work also represents the first time direct metallization and plating methods have been combined in an electrospun nanowire TCE.

## Results and discussion

Our nanowire TCE fabrication process focuses on performance and simplified processing. Though prior reports have demonstrated copper plating on silver nanowire seed layers^41,42^, the present work uniquely forms core–shell structures and targets TCE applications. The process leverages a recent development in direct metallization by Kiremitler et al. who incorporated reactive silver ink into an electrospinning solution^31^. Replacing traditional particle-based metal inks with reactive inks for direct metallization is advantageous for several reasons, including: (1) higher ink stability, (2) less expensive ink synthesis, and (3) fewer ink contaminants^43–45^. Steps 1 and 2 in Fig. 1b represent the entire Kiremitler et al. process, which is very simple but produces relatively low FOMs (171.6 × 10^–3^ Ω^−1^).

We hypothesized that junction resistances were limiting the performance, which an additional electroless deposition step could remedy. This extension to the Kiremitler et al. process resulted in a ~ 2.8 × higher FOM while also increasing the nanowire tunability (Fig. 1a). We chose copper as the electroless plating metal because it is inexpensive and highly conductive. However, other plating metals like silver or nickel should also be effective. The core–shell structure is a result of the two different metallization methods. Ideally, we would electrolessly deposit copper onto the silver nanowires immediately after calcination, but we found that a surface activation step was necessary for more uniform plating (Fig. S1). We chose UV-ozone for the activation step because it is a low-cost and non-vacuum process for improved manufacturability.

**Figure 2:**
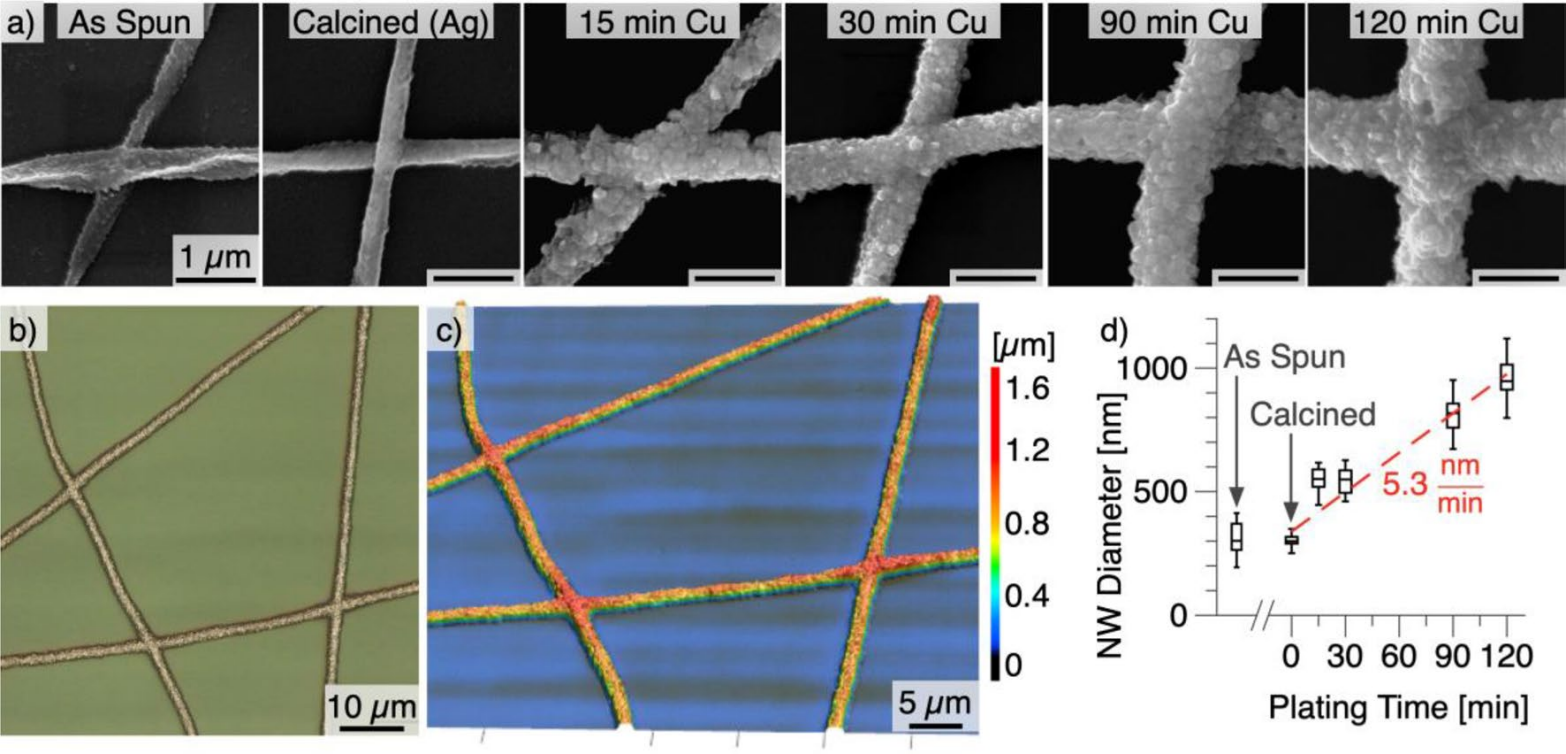
(**a**) SEM images showing nanowire junctions after electrospinning, calcination, and electroless copper plating for 15–120 min. (**b**) Optical image of a sample after copper plating shows three uniformly fused junctions. (**c**) Three-dimensional height map of nanowires after copper plating. (**d**) Plot of nanowire diameter versus plating time. The red line is a linear regression of the data.

Electroless plating contributes to lower R_s_ in two ways: (1) decreasing the resistance of individual nanowires by increasing their current carrying cross-section and (2) decreasing the contact resistances between nanowires by fusing the junctions. SEM images of nanowire junctions illustrate the junction fusing capability of copper plating (Fig. 2a). The calcined SEM image shows individually stacked silver nanowires with unfused junctions. The copper plating uniformly coats all surfaces and provides a low-resistance path between nanowires. This allows electrons to move through the silver core and copper shell of an individual wire, then transfer between wires through the copper shell. Importantly, all junctions fuse uniformly after copper plating (Fig. 2b). The final nanowires have a width-to-height ratio of ~ 1 (Fig. 2c), with slightly raised junctions due to the stacked wire starting condition (Fig. S2).

**Figure 3:**
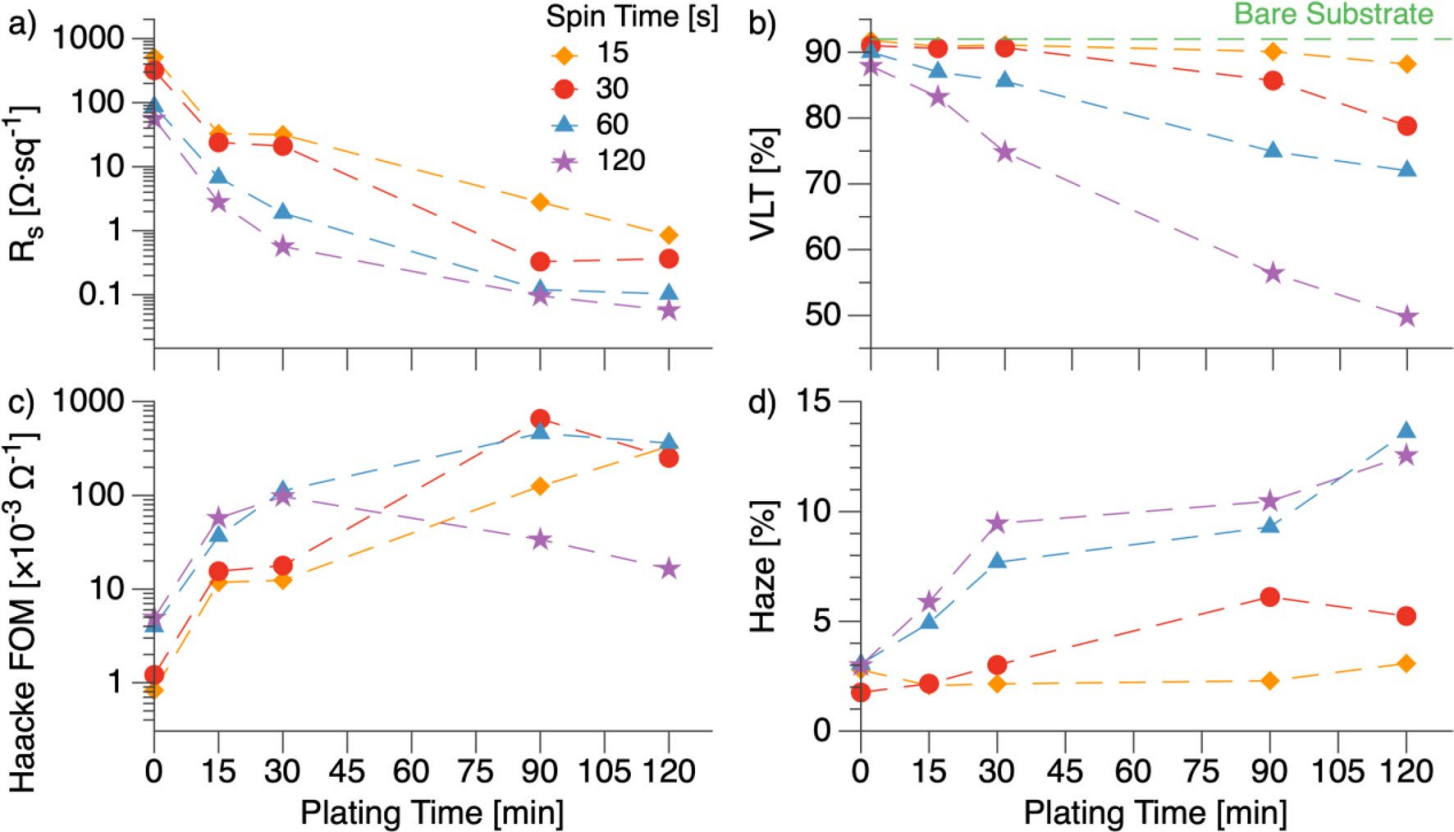
Electrospinning and electroless deposition parameter space investigation. (**a**) Sheet resistance (R_s_) versus copper plating time. (**b**) Visible Light Transmittance (VLT) versus copper plating time. The dashed green line represents the transmittance of the bare glass substrate. (**c**) Haacke figure of merit (FOM) versus copper plating time. (**d**) Optical haze versus plating time.

Comparing the nanowire growth rate to the decrease in R_s_ provides evidence that junction fusing is the main contribution of copper plating (Fig. 3a). The formula for resistance of a single nanowire is: r = ρ L A^−1^, where *r* is the resistance, *ρ* is the material resistivity, *L* is the length, and *A* is the cross-sectional area. If increasing area were the primary contribution, we would expect a one-to-one relationship between area increase and R_s_ decrease. However, from calcination to 120 min of copper plating, nanowire cross-sectional area increased by ~ 10 × (diameter increased from ~ 300 to ~ 950 nm, Fig. 2d), whereas R_s_ decreased by ~ 700 × (244–0.34 Ω sq^−1^, Fig. 3a). The significant R_s_ decrease compared to the cross-sectional area increase indicates that cross-sectional area is a minimal contribution of copper plating.

Electrospinning and electroless plating duration are two of the most impactful fabrication parameters for the Ag–Cu nanowires used in this study. Electrospinning time affects the network density, whereas the plating duration affects the individual wire thicknesses and junction resistances. At the extreme limit, a TCE with low wire density and high plating time can be conceptualized as a single nanowire spanning the substrate, and a TCE with high density and low plating time can be thought of as a continuous thin silver film. The former TCE will have visible wires and poor uniformity, whereas the latter will have low transmittance since all light must pass through a metal layer. To find the optimal conditions for this system, we performed a parameter space investigation with four different electrospinning durations (15–120 s) and five different continuous plating times (0–120 min) (Fig. 3, Table S2). Plating time was limited to 120 min because the copper plating solution homogeneously nucleated copper particles that physisorbed onto the substrate when longer plating times were attempted.

As expected, greater electrospinning times or plating times yielded lower R_s_ and visible light transmittance (VLT) values. R_s_ decreased approximately exponentially with plating time, whereas VLT decreased with a near-linear trend in time (Fig. 3a, b). The non-linear R_s_ decrease shows how junction fusing provided diminishing performance returns. Initially, copper plating rapidly reduced R_s_ by providing new pathways for electrons to travel. Once the junctions were sufficiently fused, additional plating only improved R_s_ by increasing the wire’s current carrying cross-sectional area. Larger wires also blocked more photons and decreased VLT. Indeed the linear wire diameter growth rate (Fig. 2d) correlates well with the linear VLT decrease. It is important to note that we report transmittance as VLT, whereas most publications report the transmittance at 550 nm (T_550_, the wavelength of maximum human eye sensitivity). VLT is advantageous because it weights the entire transmission spectra to the human eye’s sensitivity^3,46^. Unlike many thin-film oxide-based TCEs, which have wavelength-dependent features due to doping or interference, the transmission spectra of our Ag–Cu core–shell nanowires are nearly flat across the visible spectrum (Fig. S3). As a result, the VLT and T_550_ values were within 1% for all samples. The flat transmission spectra also result in a color-neutral appearance (Fig. 1c), making these TCEs applicable to aesthetic applications like displays and windows.

The Haacke FOM sharply increased upon initial copper plating due to rapid R_s_ improvement caused by junction fusing (Fig. 3c). However, the R_s_ improvement rate decreased as plating continued, and the VLT loss rate remained the same. Additional plating reduced the FOM after a critical point. Greater electrospinning times showed shorter critical plating times. The parameter space investigation yielded a maximum FOM of 652 × 10^–3^ Ω^−1^ (R_s_ = 0.33 Ω sq^−1^, VLT = 86%) at 30 s of electrospinning followed by 90 min of copper plating. The 652 × 10^–3^ Ω^−1^ FOM is a state-of-the-art result for nanowire TCEs, which was fabricated using all-atmospheric fabrication processes and inexpensive materials.

In addition to R_s_, VLT, and FOM, haze is an important consideration for TCEs. Low haze (< 3%) is desired for displays and window applications. The haze of Ag–Cu core–shell nanowire arrays tended to increase with greater plating and electrospinning times (Fig. 3d). After calcination, all samples had ≤ 3% haze. After copper plating, the haze increased to a maximum of 3.1, 6.1, 13.6, and 12.6% for samples with 15, 30, 60, and 120 s of electrospinning, respectively. Metal nanowire TCEs are known to exhibit high haze because sub-wavelength features strongly scatter light^47^. Strategies exist to reduce haze in metal nanowire TCEs^48^. However, in some applications like photovoltaics, high haze is desirable. Solar cells benefit from top electrodes with high haze because the scattered light increases absorption in the active layer and leads to increased efficiency^16,49^.

Flexibility is a key advantage of nanowire networks over ITO. ITO performance significantly degrades under bending due to cracks that form under small strains (< 2%)^50^. These cracks propagate and grow as strain or bending cycles increase^51^. On the other hand, metal nanowire networks show excellent bending durability^16,52,53^ due to the ductility of metals and reduced dislocation nucleation and accumulation in nanowires^54,55^.

**Figure 4:**
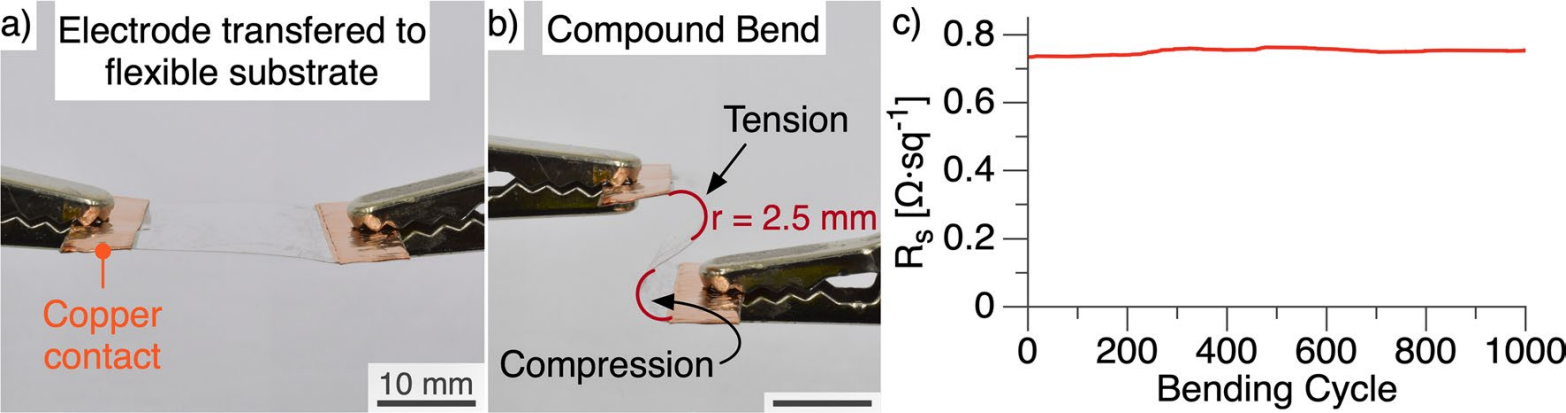
Mechanical durability evaluation. (**a**) Photograph of electrode after transferring onto a polypropylene substrate. (**b**) Photograph of TCE under 2.5 mm radius compound bending. Arrows indicate which areas are under compression and tension. (**c**) Sheet resistance (R_s_) as a function of bending cycles. Sheet resistance increased by 2.5% after 1000 bending cycles.

We evaluated bending durability of our Ag–Cu core–shell nanowire TCEs by transferring the electrodes onto flexible substrates and conducting bending experiments. The transfer process used clear polypropylene tape to delaminate the electrodes from the glass substrate. Glass substrates that were rigorously cleaned (see Methods) failed to allow delamination of the electrodes due to the strong adhesion between the electrodes and glass. The electrodes showed weaker adhesion to uncleaned glass substrates and enabled the tape transfer process (Fig. 4a). The transferred electrode was subjected to ~ 2.5 mm radius compound bending cycles (Fig. 4b). The compound bend allowed us to evaluate the TCE under compression and tension simultaneously. The resistance increased from 734 to 753 mΩ sq^−1^ after 1000 bending cycles, which represents a small 2.5% increase in sheet resistance and high bending tolerance (Figs. 4c, S4). 2.5% increase in sheet resistance is comparable to the state-of-the-art for nanowire electrodes under similar bending conditions (Table S3)^27,34,39,56–59^.

Chemical durability is a concern for metal nanowire TCEs, especially copper, which is known to oxidize in air. Nanosized metals are reactive in atmospheric oxygen and water because of their large surface-to-volume ratios. Our nanowires and others^33,34,60^ are between 500 and 1000 nm, which is larger than many literature examples of < 100 nm^29,61–63^. The larger diameter of our materials yields enhanced chemical durability. For instance, 20–40 nm copper nanowires show a 63% increase in sheet resistance after 140 h in air^64^. Atmospheric degradation of our Ag–Cu core–shell TCEs was investigated by measuring the R_s_ of each sample six months after fabrication (Table S2). Our > 500 nm diameter nanowires perform significantly better than smaller diameter nanowire electrodes with a typical R_s_ increase of 4% or less after > 4000 h (6 months). There are a number of strategies to improve the oxidation resistance of copper nanowire electrodes, including encapsulants^65–70^ or surface passivation^71–75^.

## Conclusion

State-of-the-art TCEs (FOM > 650 × 10^–3^ Ω^−1^) with broad applicability were fabricated using industrial processes. The novel direct metallization and plating approach begins with electrospinning a polymer and reactive silver ink solution to form silver nanowires. The silver nanowires show high R_s_ values due to high junction resistances. This work uniquely uses silver nanowires for two roles: (1) as an inexpensive catalyst for electroless copper deposition during device fabrication and (2) to conduct electrical current during device operation. We showed conformal copper coating drastically improved R_s_ by fusing wire junctions and not by increasing the current carrying area. These Ag–Cu nanowires show class-leading R_s_ and T while avoiding expensive materials like palladium. Furthermore, adjusting the electrospinning time or the copper deposition duration tunes TCE properties. The high-performance TCE was fabricated using simple processing and demonstrated high chemical and mechanical durability.

## Methods

### Electrospinning solution preparation

We synthesized the electrospinning solution based on the procedure developed by Kiremitler et al.^31^. The ink has three main constituents: reactive silver ink, two polymers, and water and ethanol as solvents. We made the reactive silver ink and polymer solution separately, then combined them before electrospinning. We made the reactive silver ink following the ink synthesis procedure developed by Walker and Lewis^43^ and used all chemicals without further purification. First, we weighed 2.00 g of silver acetate (anhydrous 99%, Alfa Aesar) using an analytical balance and added it to a plastic test tube. Next, we added 5.0 ml of ammonium hydroxide (28–30%, ACS grade, Sigma Aldrich) and vortex mixed until the salt was completely dissolved. We then added 0.40 ml of formic acid (≥ 98%, ACS grade, Sigma Aldrich) dropwise over 60 s, vortex mixing between drops. After synthesis, we let the test tubes sit at room temperature for 12 h in a dark environment before filtering the supernatant through a 450 nm syringe filter and storing them at 4 °C until use. We made the polymer solution by mixing 40.0 mg poly(ethylene oxide) (PEO, 900.0 kg/mol Sigma-Aldrich), 50.0 mg polyvinylpyrrolidone (PVP, 1300.0 kg/mol Sigma-Aldrich), 0.5 ml ethanol (100%, Fisher Scientific), and 0.5 ml deionized water in a capped syringe. We used all chemicals as received without further purification. We vortex mixed the syringe for 2 h, then let the solution sit for at least 24 h to allow the polymers to dissolve fully. On the day of electrospinning, we added 1.5 ml of the reactive ink to the syringe mixture and vortex mixed for 30 min. Then we inverted the syringe and let all bubbles rise to the top before attaching a needle to the syringe for electrospinning. By making the solution in the same syringe used for electrospinning, we avoided the possibility of losing constituents when transferring the small volumes between different containers.

### Electrospinning process

We used a lab-built electrospinner (Fig. S5) that consisted of a syringe pump (New Era Pump Systems NE-1010), rotating collector plate (100 rpm), and high voltage supply (ESDEMC ES813-P30.1). We connected the syringe to an 18-gauge needle and connected the needle to the positive output of the high-voltage supply with an alligator clip. We used a flow rate of 0.2 ml/hr, a needle-to-collector distance of 17 cm, and a voltage of 15 kV. The electrospinning duration varied from 15 to 120 s to achieve different wire densities. The substrates were microscope cover glasses (22 mm × 22 mm × 150 µm). Before electrospinning, substrates were cleaned with a 15 min sonication in acetone, then isopropanol, followed by 10 min of UV-ozone cleaning (Setcas, SC-UV-1).

### Calcination and silver surface activation

Immediately after electrospinning, we placed each sample on a hotplate at 300 °C for 30 s. Then immediately before electroless deposition, each sample received a 10 s UV-ozone treatment (Setcas, SC-UV-1).

### Electroless copper deposition

We synthesized an electroless copper plating solution following a recipe developed by Ben Krasnow^76^. We used all chemicals as received and ensured the previous chemical was fully dissolved before adding the next chemical. First, we added 300 mL of DI water to a beaker with a stir bar and brought it to 40 °C using a hotplate. Then, in order, we added 6.00 g of potassium sodium tartrate tetrahydrate (ACS reagent, 99%, Sigma-Aldrich), 1.50 g copper (II) sulfate pentahydrate (99.995%, Sigma-Aldrich), 1.20 g sodium hydroxide (ACS reagent, ≥ 97%, Sigma-Aldrich), 1.50 g sodium carbonate (ReagentPlus®, ≥ 99.5 Sigma-Aldrich), and 3 ml formaldehyde (ACS reagent, 37 wt.% in H_2_O, containing 10–15% Methanol as stabilizer, Sigma-Aldrich). During plating, polyimide tape suspended the samples in the solution. A hotplate maintained the bath at 35 °C with no stirring. The deposition time varied from 15 to 120 min. After copper plating, we submerged the samples in two consecutive beakers of DI water to remove excess solution, then blow-dried using compressed nitrogen. We used separate samples for each data point because resubmerging samples in the copper plating solution tended to cause wire delamination.

### Characterization

We measured light transmittance with a visible spectrophotometer (Thermo Scientific, GENESYS 40) over the wavelength range of 325–1100 nm in 1 nm increments. We blanked the spectrophotometer with air, so all transmittance values include the substrate contribution. A custom Python script converted the transmittance spectra to Visible Light Transmittance (VLT). VLT provides a single transmittance value that weights each wavelength according to the human eye's sensitivity. The VLT value was always within 1% of the transmittance at 550 nm. A Varian Cary 5G UV–Vis-NIR Spectrophotometer with an integrating sphere measured the optical haze.

We obtained the sheet resistance by measuring the resistance between two pads separating a square area of TCE material^60,77^. After fabricating the TCEs, we created silver probing pads along two opposing sides of the samples by airbrushing silver paint, then a 100 °C hotplate cured the silver for ~ 1 min. We removed excess wires so that the pads encompassed an 18 mm × 18 mm square area. We used a source measurement unit (Keysight, B2901A) and a 4-point probe station to collect current–voltage data over the range of 0–10 mA. We calculated the sheet resistance, R_s_, using the inverse of the current–voltage slope. Each sample showed an ohmic current–voltage response (Fig. S6).

Bending samples were delaminated using polypropylene tape (Duck® Brand HD Clear™, 66 µm thick). Copper tape contacts, separating an 18 mm × 18 mm square area of the TCE material, were added on opposing sides of the sample (Fig. S4). Alligator clips, connected to the copper contacts, served as connection points to apply bending and measure sheet resistance in situ. A Nordson Pro4L robot pushed and pulled the alligator clips to achieve a 2.5 mm compound bend. Calipers were used to measure the bending diameter at the red arcs indicated in Fig. 4b. An Agilent 34461A digital multimeter, connected to the alligator clips, continually monitored the sheet resistance throughout the bending cycles.

Morphology images were collected with a scanning electron microscope (SEM, Amray 3300 with SEMView 8000 electronics). We used 7 kV accelerating voltage and coated the samples with gold prior to imaging. A laser microscope (Keyence VK-X260K) collected the 3D profile measurements.

## Supplementary Information

Supplementary Information.

## Data Availability

The datasets used and/or analyzed during the current study are available from the corresponding author upon reasonable request.
