# Supplementary material for: All-atmospheric fabrication of Ag–Cu core–shell nanowire transparent electrodes with Haacke figure of merit >600 × 10–3 Ω−1
Source: Sci Rep. 2022 Dec 5;12:20962. doi: 10.1038/s41598-022-25080-x (PMC9722900; doi:10.1038/s41598-022-25080-x)
Supplement: Supplementary file 1 — Supplementary Information. [file 41598_2022_25080_MOESM1_ESM.pdf]

Supporting Information

for

All-Atmospheric Fabrication of Ag-Cu Core-Shell Nanowire Transparent Electrodes with Haacke Figure of Merit >600 × $10^{-3}\cdot\Omega^{-1}$

Steven J. DiGregorio[ab], Collin E. Miller[b], Kevin J. Prince[ab], Owen J. Hildreth[b], Lance M. Wheeler[a*]

[a]National Renewable Energy Laboratory
Golden, CO 80401 USA

[b]Colorado School of Mines
Golden, CO 80401 USA

*Correspondence: Lance.Wheeler@nrel.gov

May 2022

Table of Contents

1. UV-Ozone Surface Treatment ... 2
2. Calculating an Effective FOM for TCEs Reported in the Literature ... 3
3. Low Magnification SEM Images ... 4
4. Flat Transmittance Spectra ... 5
5. Parameter Space Investigation Raw Data ... 6
6. Additional Bending Experiment Information ... 7
7. Electrospinner Setup ... 9
8. Ohmic IV Response ... 10
References ... 11

1. UV-Ozone Surface Treatment

A primary aspect of the TCE fabrication process is electroless copper deposition onto silver nanowires to fuse the junctions. We found that the plating was inconsistent, and the wires tended to delaminate if we skipped a surface cleaning step. Preliminary studies showed that UV-ozone treatment before electroless deposition could increase the deposition rate and uniformity. Figure S1 shows the impact of different UV-ozone durations (0 s, 10 s, and 100 s) on the sheet resistance (R_s), visible light transmittance (VLT), and figure of merit (FOM) as plating time increases. The samples without surface treatment did not follow any consistent trends due to inconsistent plating. The 10 s and 100 s UV-ozone times increased the plating rate and resulted in uniform trends. We chose 10 s of UV-ozone treatment because it resulted in the highest FOM.

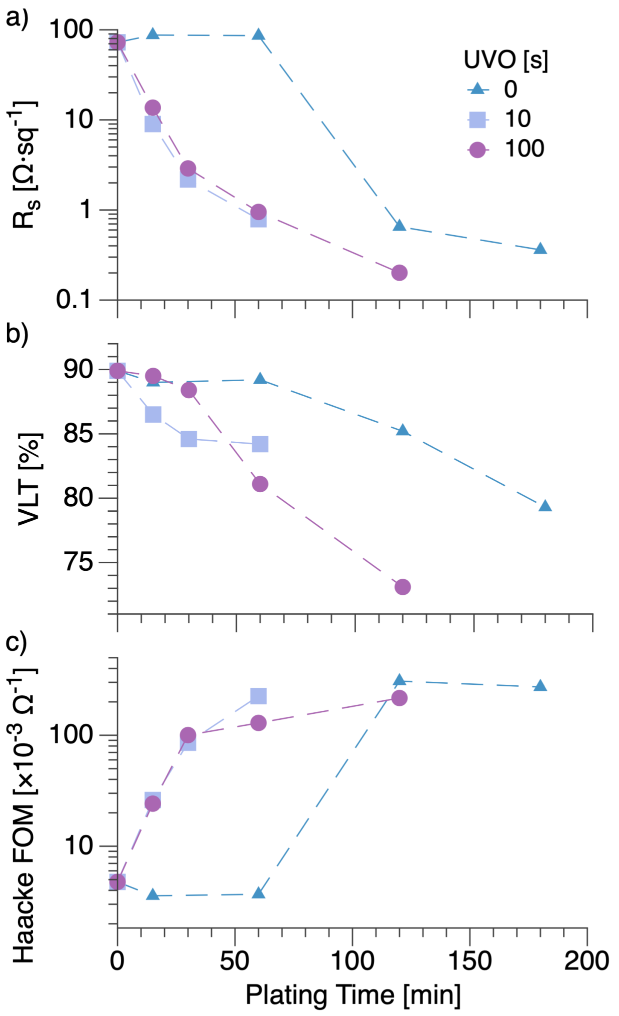

Figure S1. Impact of different UV-ozone treatment times on the Rs, VLT, and FOM. 10 s of UV-ozone treatment resulted in consistent plating and the highest FOM.

2. Calculating an Effective FOM for TCEs Reported in the Literature

Figure 1a in the main text compares several of the highest reported Haacke Figure of Merit (FOM) nanowire TCEs.[1–9] For each TCE, we recalculated the FOM based on the best R_s and Transmittance (T) reported in the original publication using the Haacke FOM equation: $T^{10} \cdot R_s^{-1}$. Some papers specifically mentioned that they removed the effect of the substrate.[4,8,9] In those cases, we multiplied the reported transmittance value by 0.92 before calculating the FOM. This essentially added a 92% transmittance substrate, like the ones used in this work, in series with the TCE.

Table S1. Data for the highest FOM nanowire TCEs in the literature. These data were used to create Figure1a in the main text. *Author specified that the reported transmittance value does not include the effect of the substrate. In these cases, the transmittance value in the table is an effective transmittance calculated by multiplying the reported percent transmittance by 0.92.

Reference	R_s [$ohm \cdot sq^{-1}$]	T_{550} [%]	FOM (Haacke) [$\times 10^{-3} \cdot \Omega^{-1}$]	TCE type
1	10	90	34.9	ITO
2	0.03	86.5	7817.0	Polymer Imprinting
3	0.07	83	2216.6	Polymer Imprinting
4*	0.61	86.5	383.6	Polymer Imprinting
5	0.42	89.2	762.7	Electrospinning
6	2	90	174.3	Electrospinning
7	1.9	89.4	171.6	Electrospinning
8*	1.3	82.8	116.5	Electrospinning
9*	2.2	83.8	77.7	Electrospinning
This work	0.33	85.7	651.9	Electrospinning

3. Low Magnification SEM Images

Figure S2 contains lower magnification SEM images of the nanowire junctions from Figure 2 of the main text. The images show consistent wire diameters and junctions.

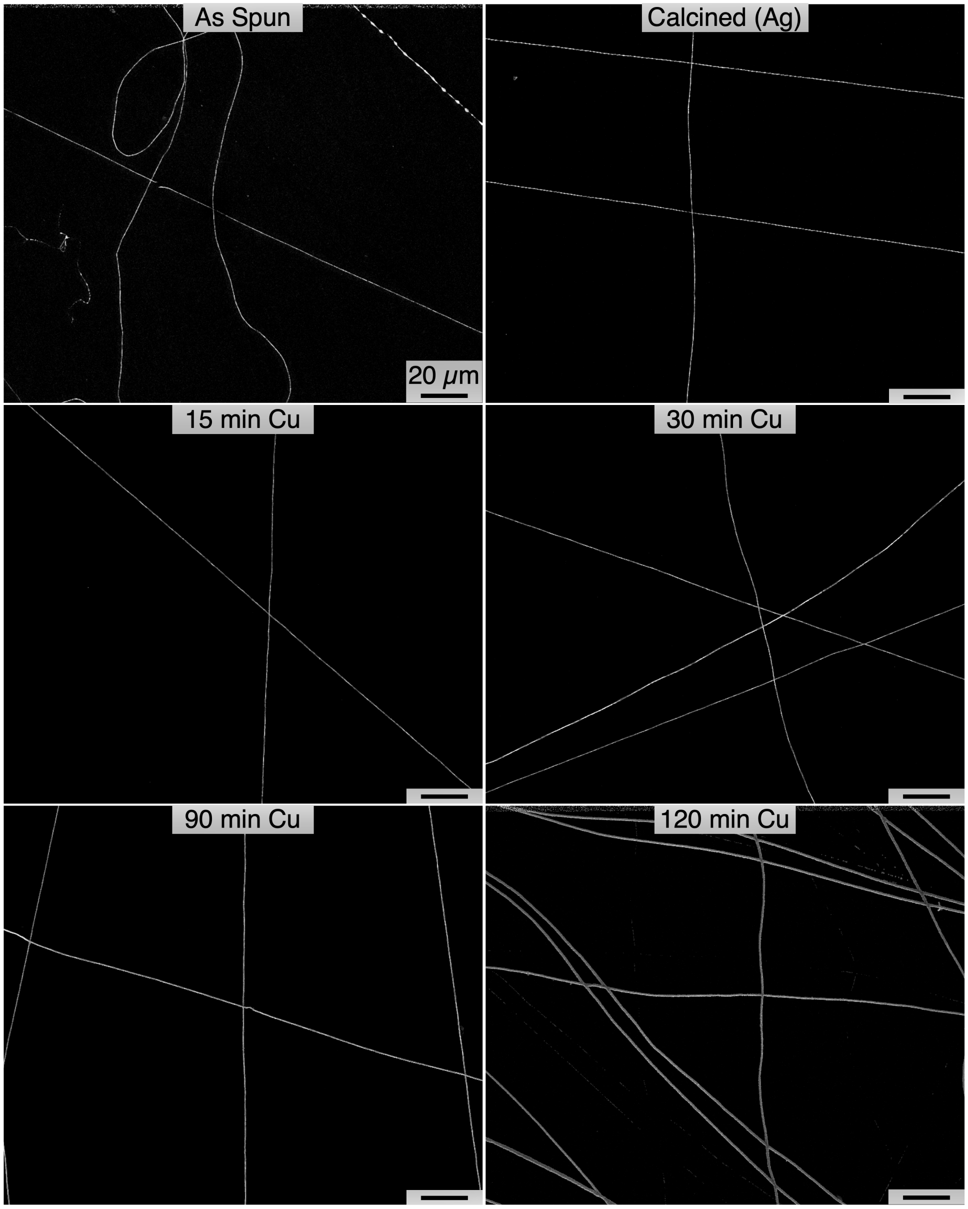

Figure S2. Low magnification SEM images that show multiple wire junctions for the as-spun, calcined, and copper plated nanowires.

4. Flat Transmittance Spectra

Figure S3 plots the full transmittance spectra for each sample in the parameter space study. There are too many curves to show in a single graph, so we separated them by plating times. The green line indicates the transmittance spectra of the glass substrates. The relatively flat transmittance spectra in the range of human vision lead to the color-neutral appearance of the TCEs.

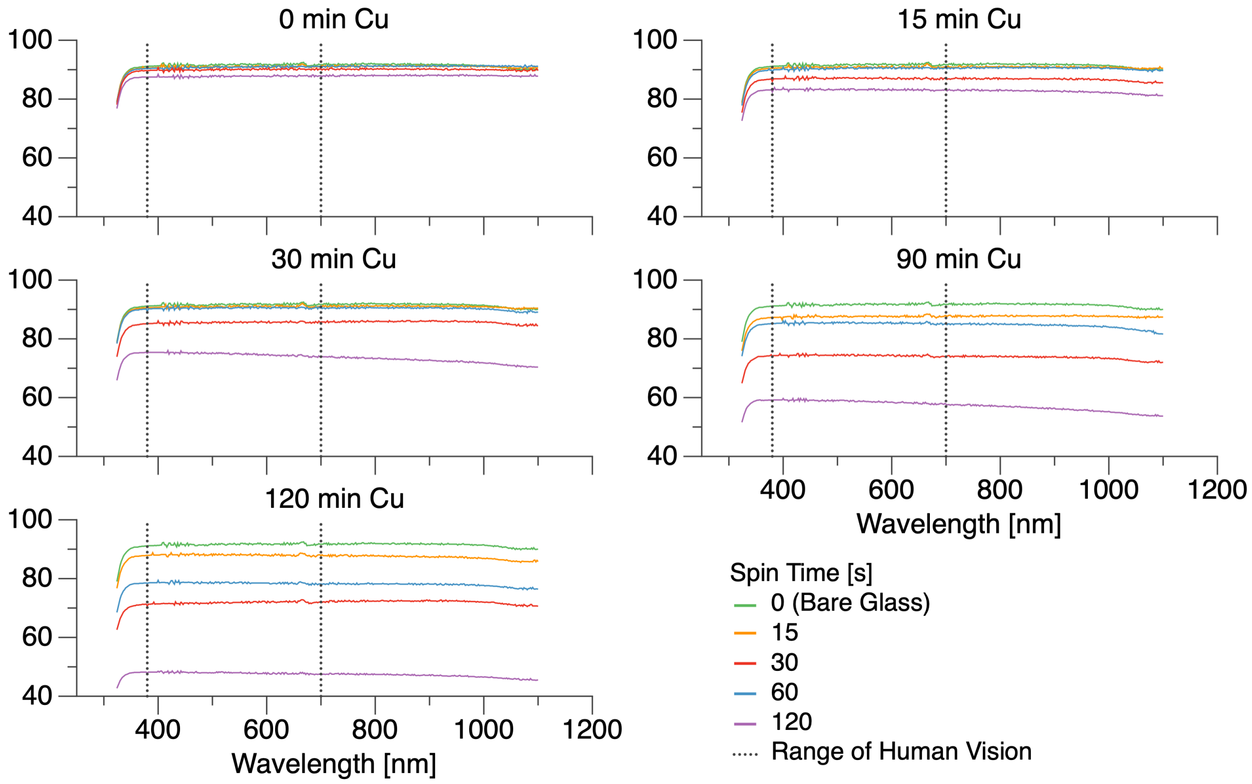

Figure S3. Full transmittance spectra of the TCEs from the electrospinning time and plating time parameter space investigation. The green line indicates the transmittance spectra of the bare glass substrate.

5. Parameter Space Investigation Raw Data

Table S2 contains the raw data from the spin time and plating time parameter space investigation. The table shows transmittance, haze, sheet resistance (R_s), and figure of merit measured after fabrication. The table also contains the R_s of each sample remeasured six months after fabrication to determine the impact of atmospheric degradation. R_s increased by only 4% on average over the six months.

Table S2. Raw data from the electrospinning time and copper plating time parameter space investigation. Data includes Visible Light Transmittance (VLT), optical haze, sheet resistance (R_s), R_s change after six months, and the Haacke Figure of Merit (FOM).

Spin Time [s]	Plating Time [min]	VLT [%]	Haze [%]	R_s [Ω/sq]	R_s After 6 Months [Ω/sq]	R_s Change After 6 Months [%]	Haacke FOM [$\times 10^{-3} \cdot \Omega^{-1}$]
15	0	91.8	2.79	512.247			0.828
15	15	90.9	2.07	32.757	33.524	2.34	11.808
15	30	91.1	2.15	31.558	31.870	0.99	12.413
15	90	90.1	2.29	2.806	2.830	0.86	125.619
15	120	88.2	3.08	0.850	0.903	6.25	334.290
30	0	91.0	1.76	320.106	368.559	15.14	1.211
30	15	90.6	2.16	23.910	24.334	1.77	15.500
30	30	90.7	3.01	21.033	21.306	1.30	17.817
30	90	85.7	6.11	0.329	0.346	5.00	651.935
30	120	78.8	5.24	0.366	0.387	5.81	251.824
60	0	90.0	3.06	87.721	92.488	5.43	3.983
60	15	87.0	4.92	6.752	6.912	2.37	36.835
60	30	85.6	9.46	1.888	1.927	2.09	112.228
60	90	74.9	9.30	0.120	0.121	0.74	460.953
60	120	72.0	13.62	0.103	0.116	11.86	362.561
120	0	87.9	2.99	55.736	58.000	4.06	4.934
120	15	83.2	5.88	2.791	2.853	2.26	57.273
120	30	74.8	7.69	0.566	0.566	0.01	97.511
120	90	56.4	10.47	0.096	0.103	6.54	33.788
120	120	49.8	12.56	0.057	0.058	2.33	16.455

6. Additional Bending Experiment Information

Figure S4a shows the magnified sheet resistance (R_s) vs. bending graph from Figure 4c in the main text. The sheet resistance started at 734 m$\Omega \cdot sq^{-1}$ and increased by a maximum of 4% after 500 bending cycles, then recovered slightly to an increase of 2.5% after 1000 bending cycles. The sheet resistance increase of 2.5-4% compares favorably to other examples of nanowire bending experiments (Table S3). We initially expected the sheet resistance to increase steadily with number of cycles. However, although the R_s trended upwards overall, the sheet resistance decreased by 1-2% at certain points in the experiment. This result is consistent with other nanowire network bending experiments, which show R_s fluctuations of a few percent over the bending tests.[3,4,10] Unlike our manuscript, the aforementioned publications did not provide a magnified graph or mention the fluctuations for us to compare directly. But we estimated that their fluctuations were greater than or equal to ours based on their full-scale graphs.

Figure S4b shows a schematic of the experimental setup for the bending experiments. After delaminating the TCE material onto flexible polypropylene tape, conductive copper tape contacts were added on opposing sides of the sample to encompass an 18 mm × 18 mm square area so that measuring the resistance between the copper contacts gave sheet resistance. Alligator clips connected to the copper contacts were connected to non-conductive stands to actuate the bending motion. One stand was fixed in position, while the other stand moved on a robotic stage for precise and repeatable bending. The alligator clips were connected to a Digital Multimeter (DMM) to monitor the sheet resistance.

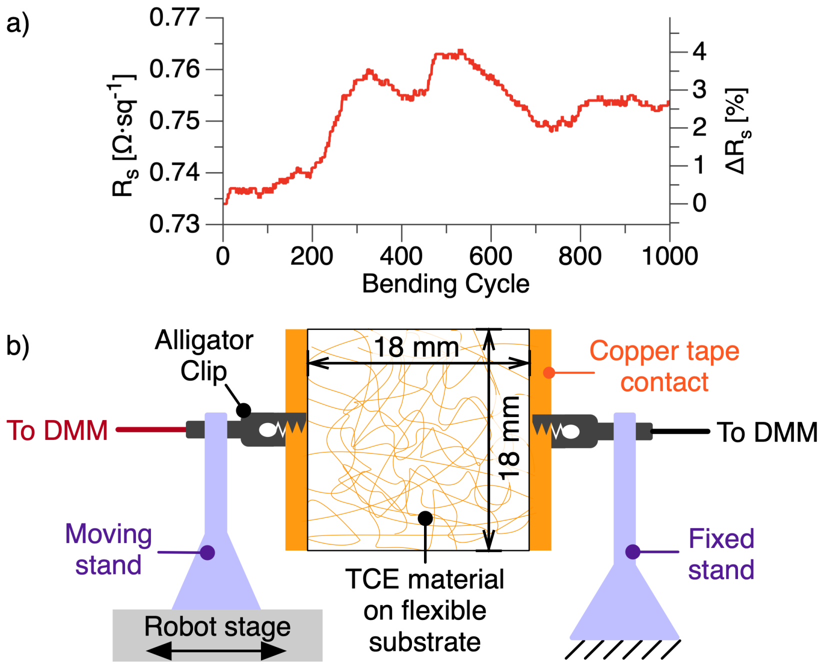

Figure S4. a) Magnified graph of Rs vs. bending cycle. Sheet resistance increased by 4% after 500 bending cycles, then recovered slightly to 2.5% after 1000 cycles. b) Schematic of bending experimental setup. Alligator clips attached to copper tape contacts were used for in situ sheet resistance measurements with a Digital Multimeter (DMM). The alligator clips were attached to a fixed and moving stand to actuate the bending cycles.

Table S3. Comparison of sheet resistance, $\Delta R_s = (R_{s,final} - R_{s,inital}) / R_{s,initial} \cdot 100$, after 1000 bending cycles for some relevant nanowire electrode literature.

Reference	ΔR_s [%]	Bending radius [mm]
Ref.[11]	1.6	0.5
Ref.[12]	2	4
This work	2.5	2.5
Ref.[13]	3	4
Ref.[9]	5	10
Ref.[10]	15	15
Ref.[14]	20	4
Ref.[4]	30	5
Ref.[15]	40	3

7. Electrospinner Setup

Figure S5 shows the lab-built electrospinner setup used in this study. The main components include a syringe pump (New Era Pump Systems NE-1010) and a high-voltage supply (ESDEMC ES813-P30.1). Samples were attached to an acrylic sample stage located in front of the collector plate. While electrospinning, a handheld drill rotated the sample stage at ~100 rpm to increase nanowire uniformity.

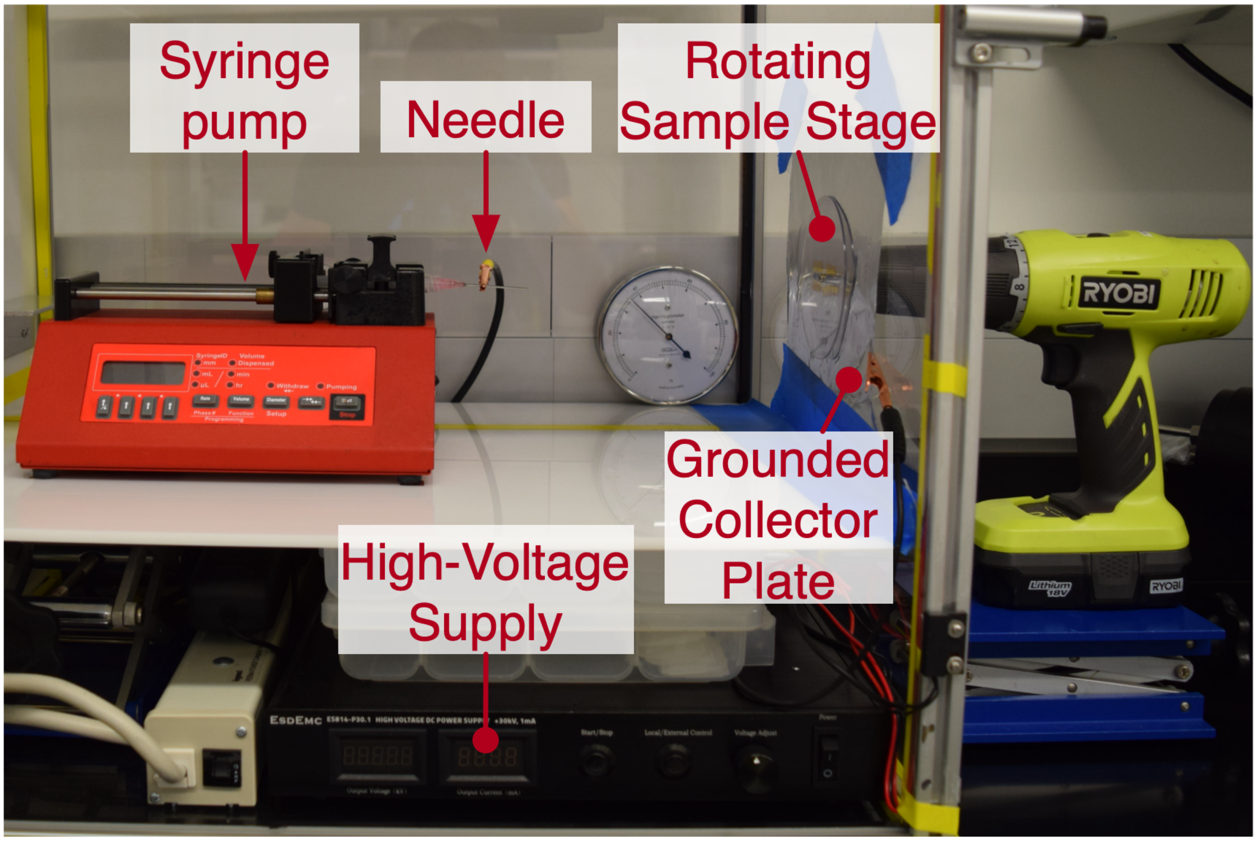

Figure S5. Image of lab-built electrospinner setup. A high-voltage supply creates an electric field between the needle and aluminum foil collector plate. A rotating sample stage in front of the collector plate increases nanowire uniformity.

8. Ohmic IV Response

Figure S6 plots the current vs. voltage (IV) curves for each sample from the parameter space study. Each curve consists of 20 data points. The linearity of the IV curves indicates an ohmic response. We calculated sheet resistance from these curves by finding the inverse of the slope.

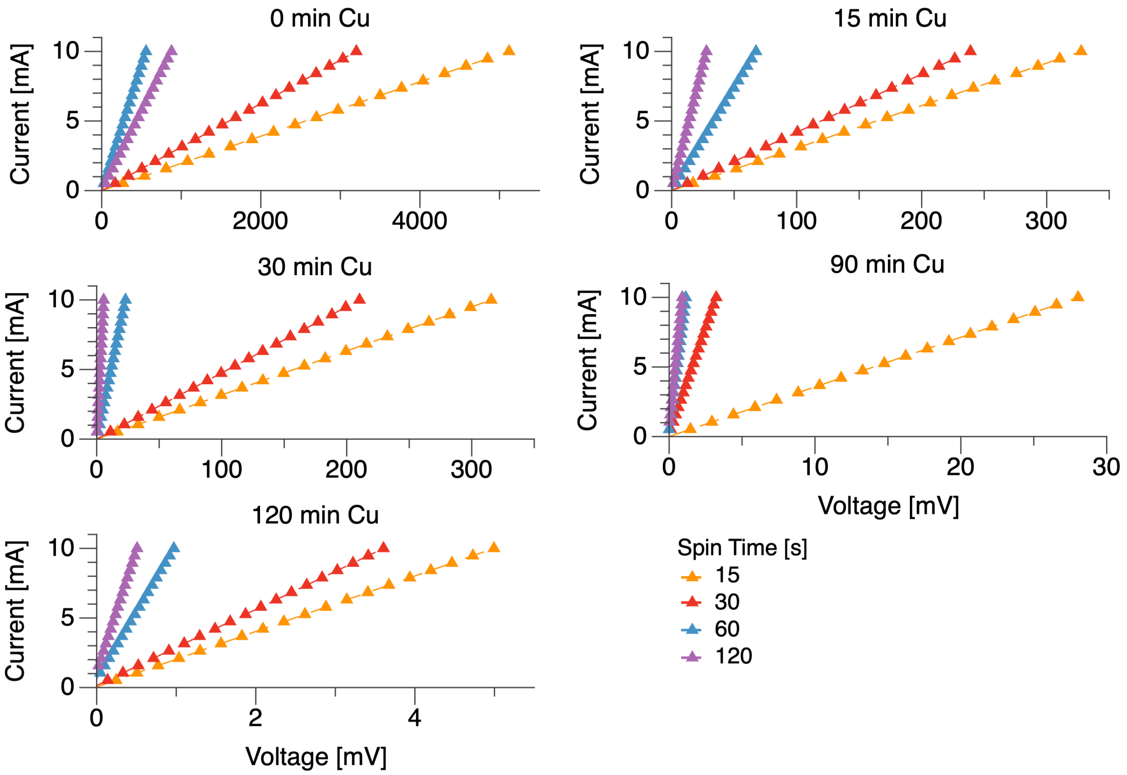

Figure S6. IV curves for each sample. The linear voltage and current relationship indicates that these electrodes have an ohmic behavior.

References

1. De, S. & Coleman, J. N. Are There Fundamental Limitations on the Sheet Resistance and Transmittance of Thin Graphene Films? *ACS Nano* **4**, 2713–2720 (2010).
2. Chen, X. *et al.* Printable High-Aspect Ratio and High-Resolution Cu Grid Flexible Transparent Conductive Film with Figure of Merit over 80 000. *Adv. Electron. Mater.* **5**, 1800991 (2019).
3. Shen, S., Chen, S.-Y., Zhang, D.-Y. & Liu, Y.-H. High-performance composite Ag-Ni mesh based flexible transparent conductive film as multifunctional devices. *Opt. Express* **26**, 27545 (2018).
4. Khan, A. *et al.* High-Performance Flexible Transparent Electrode with an Embedded Metal Mesh Fabricated by Cost-Effective Solution Process. *Small* **12**, 3021–3030 (2016).
5. An, S. *et al.* Self-Junctioned Copper Nanofiber Transparent Flexible Conducting Film via Electrospinning and Electroplating. *Adv. Mater.* **28**, 7149–7154 (2016).
6. Wu, H. *et al.* A transparent electrode based on a metal nanotrough network. *Nature Nanotech* **8**, 421–425 (2013).
7. Kiremitler, N. B., Esidir, A., Gozutok, Z., Ozdemir, A. T. & Onses, M. S. Rapid fabrication of high-performance transparent electrodes by electrospinning of reactive silver ink containing nanofibers. *Journal of Industrial and Engineering Chemistry* (2020) doi:10.1016/j.jiec.2020.12.010.
8. Jang, J. *et al.* Rapid production of large-area, transparent and stretchable electrodes using metal nanofibers as wirelessly operated wearable heaters. *NPG Asia Mater* **9**, e432–e432 (2017).
9. Bao, C. *et al.* *In Situ* Fabrication of Highly Conductive Metal Nanowire Networks with High Transmittance from Deep-Ultraviolet to Near-Infrared. *ACS Nano* **9**, 2502–2509 (2015).

10. Testa, A. *et al.* Transparent Flexible Electrodes Based on Junctionless Copper Nanowire Network via Selective Electroless Metallization of Electrospun Nanofibers. *J. Electrochem. Soc.* **164**, D764 (2017).
11. Pyo, K. & Kim, J.-W. Thermally stable and flexible transparent heaters based on silver nanowire-colorless polyimide composite electrode. *Current Applied Physics* **16**, 1453–1458 (2016).
12. Feng, X. *et al.* Cost-Effective Fabrication of Uniformly Aligned Silver Nanowire Microgrid-Based Transparent Electrodes with Higher than 99% Transmittance. *ACS Appl. Mater. Interfaces* **14**, 39199–39210 (2022).
13. Chae, W. H., Patil, J. J. & Grossman, J. C. Conformal Encapsulation of Silver Nanowire Transparent Electrodes by Nanosized Reduced Graphene Oxide Leading to Improved All-Round Stability. *ACS Appl. Mater. Interfaces* **14**, 34997–35009 (2022).
14. Hsu, P.-C. *et al.* Electrolessly Deposited Electrospun Metal Nanowire Transparent Electrodes. *J. Am. Chem. Soc.* **136**, 10593–10596 (2014).
15. Mou, Y. *et al.* Facile preparation of stable reactive silver ink for highly conductive and flexible electrodes. *Applied Surface Science* **475**, 75–82 (2019).
